# Supplementary material for: Region-of-interest analyses of one-dimensional biomechanical trajectories: bridging 0D and 1D theory, augmenting statistical power
Source: PeerJ. 2016 Nov 2;4:e2652. doi: 10.7717/peerj.2652 (PMC5101620; doi:10.7717/peerj.2652)
Supplement: Supplemental Information 1 [file peerj-04-2652-s001.pdf]

Appendix A Nomenclature

This Appendix proposes a set of terms and definitions for use in analyses of 1D biomechanical continua (Table A.1) and relates these terms to analogous, established terms from the neuroimaging literature (Table A.2). Note that the neuroimaging terminology cannot easily be used for 1D applications because the meaning of 'volume' is unclear for 1D data.

Table A.1: Proposed nomenclature for statistical parametric mapping (SPM) analyses of 1D continua.

Category	Term	Abbreviation	Description
Geometry	Point of interest	POI	A single position in a 1D continuum. (e.g. time = 10%)
	Region of interest	ROI	A continuous portion of a 1D continuum. (e.g. time = 20–50%).
Statistical inference	0D inference		*Inference procedures applied to 0D univariate or multivariate data which do not account for correlation amongst adjacent continuum points.
	1D inference		Inference procedures applied to univariate or multivariate 1D data which use multiple comparisons corrections to account for correlation amongst adjacent continuum points. RFT procedures are assumed unless otherwise stated.
	Random Field Theory	RFT	Provides analytical parametric solutions for probabilities associated with smooth 1D Gaussian continua, and in particular the probability that small samples of 1D Gaussian continua will produce test statistic continua that reach certain heights in particular experiments. Reference: Adler and Taylor (2007)
	Small region correction	SRC	A multiple comparisons correction across within-ROI values using (parametric) RFT or a nonparametric alternative. RFT is assumed unless otherwise stated.

General Methodology	0D analysis		Analysis of univariate or multivariate 0D data using 0D inference procedures.
	1D analysis		Analysis of univariate or multivariate 1D data using 1D inference procedures
	0D method		A method which conducts 0D analysis of 0D data.
	1D method		A method which conducts 1D analysis of 1D data.
	Statistical Parametric Mapping	SPM	A methodology for analyzing nD continua, often using classical hypothesis testing. SPM's classical hypothesis testing involves: (i) test statistic continuum computation from a set of registered nD continua, (ii) continuum smoothness estimation based on the nD gradient of the model residuals, (iii) critical threshold computation using RFT and the estimated smoothness, and (iv) probability value computation using RFT and the estimated smoothness for threshold-surviving clusters. Reference: Friston et al. (2007)
	Region of interest analysis	ROIA	A set of procedures involving (i) ROI definition and (ii) statistical analyses of ROI data.
Specific Methodology	0D metric-based ROI analysis	ROIA-0D	ROIA which conducts 0D analysis on a 0D summary metric (e.g. mean, median, maximum, etc.) that is meant to summarize or otherwise represent all values in an ROI. ROIA-0D with a local extremum summary metric (minimum or maximum) is common in the Biomechanics literature.
	1D ROI analysis	ROIA-1D	ROIA which conducts 1D analysis of an ROI's data using a small region correction (SRC). This is the focus of the main manuscript. Equivalent to 'SRC-based ROI analysis'.

*Note: Bonferroni and other corrections applied across multiple continuum nodes but which do not consider inter-node correlation are included in '0D inference'.

Table A.2: Relevant nomenclature from the neuroimaging literature. Terms without abbreviations do not explicitly appear in the literature and are instead introduced here to clarify connections to the proposed nomenclature in Table A.1.

Term	Abbreviation	Description
Region of interest	ROI	A continuous portion of an nD continuum. Also called a 'volume of interest' (VOI) in 3D.
Region of interest analysis		*0D analyses conducted on a 0D metric extracted from an ROI. Reference: Brett et al. (2002). Equivalent to 'volume of interest (VOI) analysis'.
Small volume correction	SVC	A multiple comparisons correction across within-ROI values using (parametric) RFT or a nonparametric alternative. RFT is assumed unless otherwise stated. Reference: SPM12 Manual (FIL Methods Group, Wellcome Trust Centre for Neuroimaging, University College London) http://www.fil.ion.ucl.ac.uk/spm/software/spm12/

Appendix B Field smoothness: FWHM

The full width at half maximum (FWHM) parameter describes the smoothness of 1D random fields. Most precisely, the FWHM specifies the breadth of a Gaussian kernel (Fig.B.1) which, when convolved with uncorrelated (perfectly rough) Gaussian 1D data yields smooth Gaussian 1D fields (Fig.B.2).

Random field theory (RFT) (Adler and Taylor, 2007) uses the FWHM value to describe the probabilistic behavior of smooth fields. The most important probability for classical hypothesis testing is the probability that the random fields will reach a certain height (Friston et al., 2007); setting that probability to $\alpha = 0.05$ yields the critical RFT thresholds depicted in Fig.3 (main manuscript).

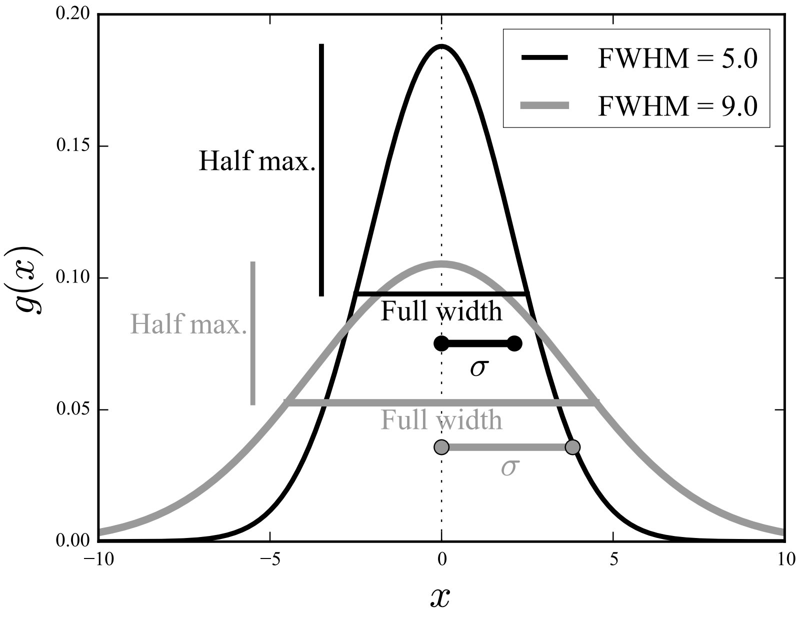

Figure B.1: Breadth parameters for Gaussian kernels: σ and FWHM.

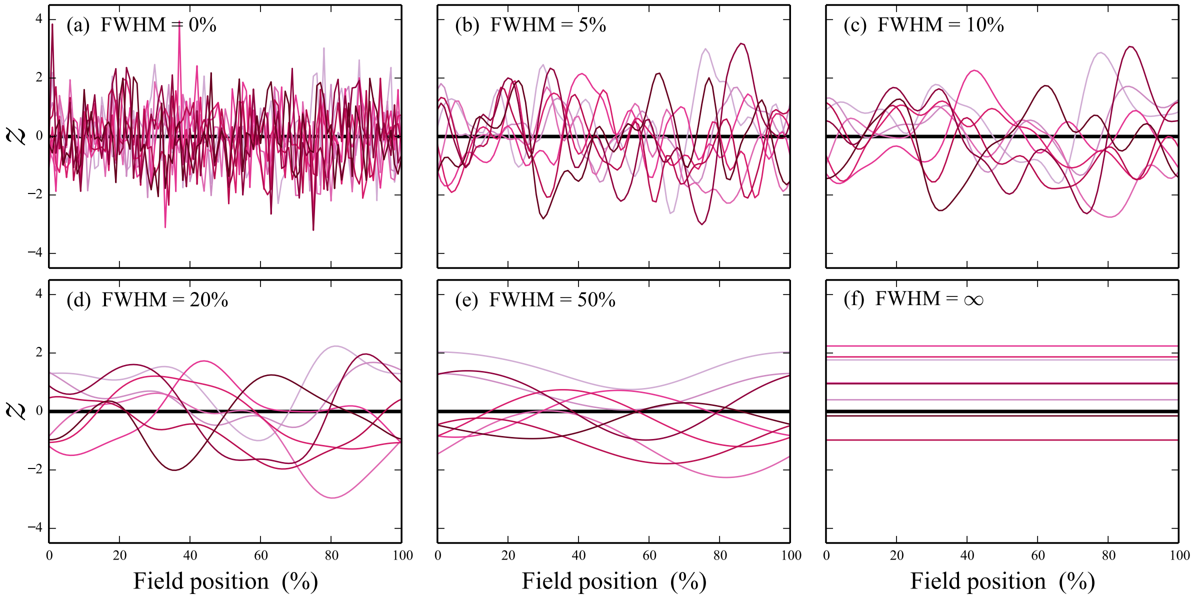

Figure B.2: One-dimensional Gaussian random fields. The FWHM parameterizes field smoothness. The smaller the FWHM the rougher the field, and the more likely random fields are to reach a specified height. Infinitely smooth fields (FWHM=∞) are probabilistically equivalent to 0D scalars. It has been shown that Biomechanics datasets generally tend to lie in the range FWHM = [5%, 50%], including (processed) kinematics, force and processed EMG (Pataky et al., 2016).

Appendix C ROI analysis in Python and MATLAB

This Appendix describes the Python and MATLAB interfaces for region of interest (ROI) analysis in **spm1d** (www.spm1d.org). Below Python and MATLAB code snippets are presented in green and orange, respectively.

C.1 Example ROI analysis

In **spm1d**, ROI analysis can be conducted using the keyword "roi" in all statistical routines from **spm1d.stats** including t tests, regression, ANOVA, etc. As an example, a two-sample t test with an ROI spanning from time = 10% to 40% can be conducted as follows:

```
import numpy as np
import spm1d

#(0) Load dataset:
YA,YB      = spm1d.data.uv1d.t2.SimulatedTwoLocalMax().get_data()


#(1) Define SR:
roi        = np.array( [False]*101 )
roi[10:40] = True


#(2) Conduct t test:
t          = spm1d.stats.ttest2(YB, YA, roi=roi)
ti         = t.inference(0.05)
ti.plot()
```

```
%(0) Load dataset:
dataset    = spm1d.data.uv1d.t2.SimulatedTwoLocalMax();
[YA,YB]    = deal(dataset.YA, dataset.YB);


%(1) Define ROI:
roi        = false( 1, 101 );
roi(11:40) = true;


%(2) Conduct t test:
t          = spm1d.stats.ttest2(YB, YA, 'roi',roi);
ti         = t.inference(0.05);
ti.plot()
```

C.2 Example analysis in detail

After importing the necessary packages, the next commands retrieve one of spm1d's built-in datasets:

```
YA,YB      = spm1d.data.uv1d.t2.SimulatedTwoLocalMax().get_data()
```

```
dataset = spm1d.data.uv1d.t2.SimulatedTwoLocalMax();
[YA,YB] = deal(dataset.YA, dataset.YB);
```

Here the variables YA and YB are both $(J \times Q)$ arrays, where J and Q are the number of observations and the number of nodes in the 1D continuum, respectively. They can be visualized as follows:

```
from matplotlib import pyplot
pyplot.plot(YA.T, color="k")
pyplot.plot(YB.T, color="r")
```

```
plot(YA', 'color','r')
hold on
plot(YB', 'color','k')
```

This produces the following figure:

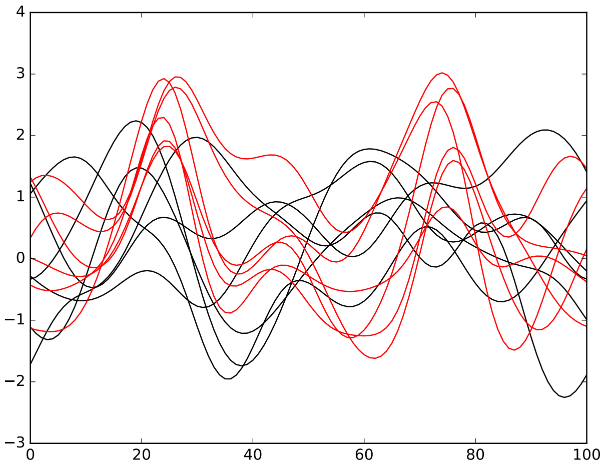

Figure C.1: Example dataset "`SimulatedTwoLocalMax`".

In this example the ROI is specified as a binary vector of length Q, where True indicates the ROI:

```
roi        = np.array( [False]*101 )
roi[10:40] = True
```

```
roi         = false( 1, 101 );
roi(11:40) = true;
```

Since the boolean values False and True numerically evaluate to 0 and 1, respectively, the ROI can be visualized using a standard plotting command (Fig.C.2a):

```
pyplot.plot(roi, color="b")
pyplot.ylim(-0.1, 1.1)
```

```
plot(roi, 'color', 'b')
ylim( [-0.1  1.1] )
```

Alternatively the ROI can be visualized using **spm1d**'s "plot_roi" function — currently only available in Python (Fig.C.2b):

```
pyplot.plot(YA.T, color="k")
pyplot.plot(YB.T, color="r")
spm1d.plot.plot_roi(roi, facecolor="b", alpha=0.3)
```

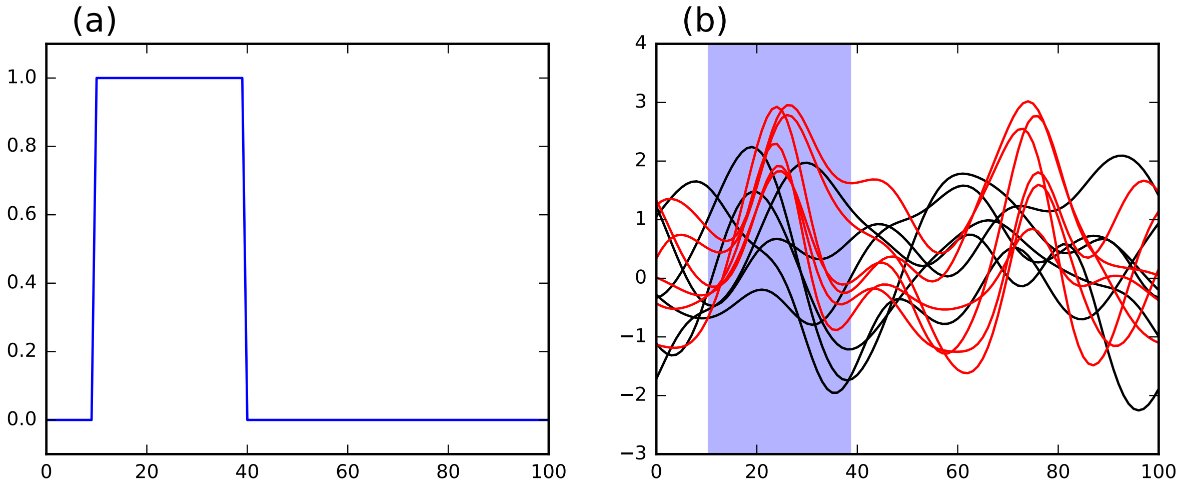

Figure C.2: Example ROI visualization (a) using `pyplot.plot` and (b) using `spm1d.plot.plot_roi`

ROI-based statistical analysis proceeds as follows (Fig.C.3a)

```
t           = spm1d.stats.ttest2(YB, YA, roi=roi)
ti          = t.inference(0.05)
ti.plot()
```

```
t          = spm1d.stats.ttest2(YB, YA, 'roi', roi)
ti         = t.inference(0.05)
ti.plot()
```

To conduct the same analysis without an ROI simply drop the "roi" keyword as follows (Fig.C.3b)

```
t          = spm1d.stats.ttest2(YB, YA)
ti         = t.inference(0.05)
ti.plot()
```

```
t          = spm1d.stats.ttest2(YB, YA)
ti         = t.inference(0.05)
ti.plot()
```

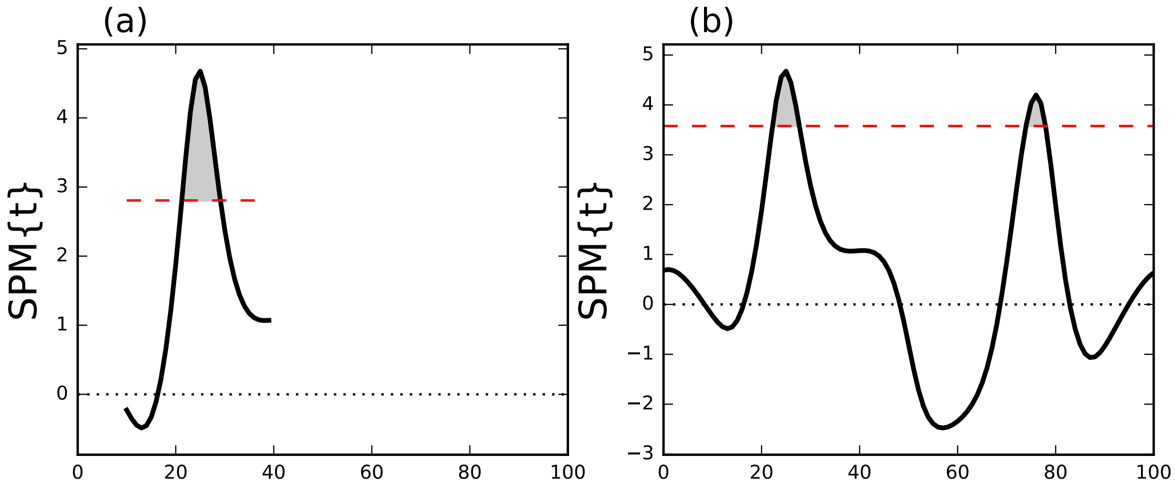

Figure C.3: Example analysis (a) with one ROI and (b) without any ROIs. "SPM{t}" denotes the t statistic extended in time to form a 'statistical parametric map'.

Note the following:

- When conducting ROI analysis **spm1d** masks portions of the t statistic curve which lie outside the specified ROIs. This masking is replicated in the statistical continuum itself (the "z" attribute of the spm object), as indicated below. Note: in MATLAB, masked elements "- -" are replaced by "NaN".

```
print( t.z )

[-- -- -- -- -- -- -- -- -- -- -0.22776187136870144 -0.35193394183457477
 -0.4454435693960439 -0.4866562443204332 -0.45343849771681227
 -0.32945352380074494 -0.10481565221034789 0.22601109469000205
```

```
0.6659607107024268 1.2186672527052544 1.8786714310449357 2.624090708123374
3.3977458417276942 4.09182563625598 4.557446151350232 4.676118446926481
4.4442233781988865 3.976243703618212 3.414764145392637 2.864043743188491
2.379655562970072 1.9818420780205368 1.6712848172222807 1.4407743903014176
1.2783724255448865 1.17159899332 1.1081081854538306 1.0770125187802424
1.0676148836585948 1.0710020774519424 -- -- -- -- -- -- -- -- -- -- -- --
-- -- -- -- -- -- -- -- -- -- -- -- -- -- -- -- -- -- -- -- -- -- -- -- --
-- -- -- -- -- -- -- -- -- -- -- -- -- -- -- -- -- -- -- -- -- -- -- --]
```

- Critical thresholds (hashed horizontal lines in Fig.C.3) are always lower in ROI vs. non-ROI analysis because the search volume is smaller.
- When conducting ROI analysis there may be regions outside of the specified ROI which would have reached significance had whole-field analysis been conducted (Fig.C.3b) and/or had the ROIs been chosen differently. This issue is discussed in the main manuscript.

C.3 Multiple ROIs in a single analysis

To conduct statistical analysis using multiple ROIs simply add multiple portions to the boolean vector:

```
roi        = np.array( [False]*101 )
roi[10:40] = True
roi[60:75] = True
```

```
roi        = false( 1, 101 );
roi(11:40) = true;
roi(61:75) = true;
```

When there are multiple ROIs **spm1d** automatically raises the critical threshold to account for the expanded search volume (Fig.C.4). Note that the critical threshold for two ROIs of breadths b_1 and b_2 is not equivalent to the the critical threshold for a single ROI of breadth ($b_1 + b_2$). For example, using the two-ROIs defined above yields a critical threshold of t^*=3.115. If, instead a single ROI of the same total breadth had been defined as follows:

```
roi        = np.array( [False]*101 )
roi[10:55] = True
```

```
roi        = false( 1, 101 );
roi(11:55) = true;
```

the critical threshold would be slightly lower: t^*=3.050. The reason is that within-ROI variance is smooth (across the 1D continuum), but between-ROI variance is generally not smooth. The **spm1d** software accounts for this using the Euler characteristic of the specified ROI (see Pataky 2016).

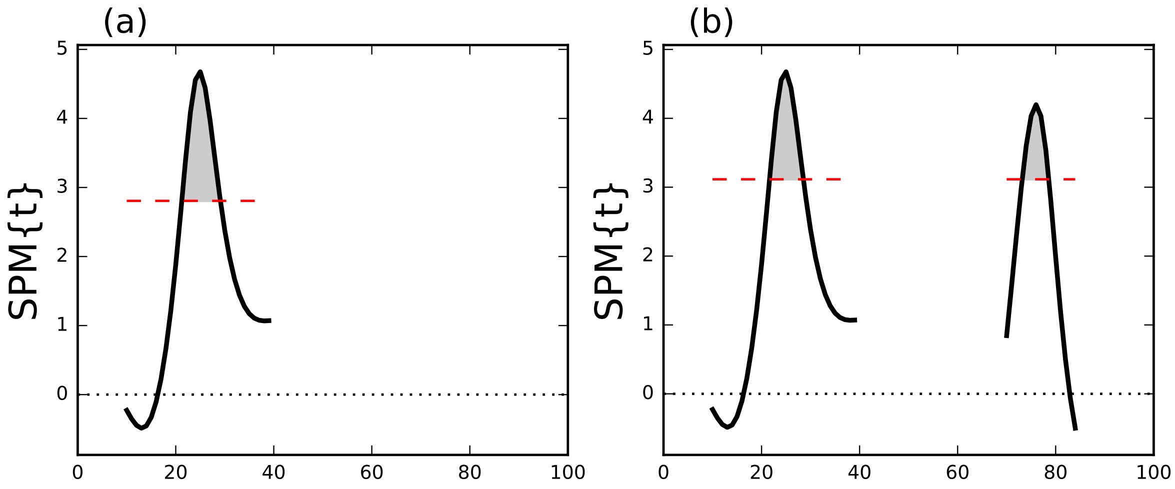

Figure C.4: Example analysis (a) with one ROI and (b) with two ROIs.

C.4 Directional ROIs

'Directional ROIs' can be used to simultaneously test a set of one-tailed hypotheses. As depicted in Fig.5 (main manuscript), different ROIs can embody one-tailed hypotheses. Directional ROIs are specified in **spm1d** as an integer vector containing the values: -1, 0 and +1 as follows:

```
roi         = np.array( [0]*101 )
roi[10:40] = +1
roi[60:75] = -1
```

```
roi         = zeros( 1, 101 );
roi(11:40) = +1;
roi(61:75) = -1;
```

Results for this particular set of directional ROIs as applied to the dataset above are depicted in Fig.C.5b. Interpreting this result is somewhat complex and warrants discussion. First let us compare this result with the case where both ROIs are positive (Fig.C.5a):

```
roi         = np.array( [0]*101 )
roi[10:40] = +1
roi[60:75] = +1
```

```
roi         = zeros( 1, 101 );
roi(11:40) = +1;
roi(61:75) = +1;
```

There are two results to consider:

1. *Omnibus result*: The omnibus result involves simultaneous testing of all ROIs. In this example there is sufficient evidence to reject the omnibus null hypothesis for both Fig.C.5a and C.5b because the SPM{t} crosses the critical threshold in the predicted direction in both cases.

2. *Individual ROI results*: These results may be regarded as a *post hoc* qualification of the omnibus test, much in the same way *post hoc* t tests work in ANOVA. In Fig.C.5a the null hypotheses are rejected for both ROIs, but in Fig.C.5b the null hypothesis is rejected only for the first ROI.

In other words, there is sufficient evidence to reject the omnibus null hypothesis for both Fig.C.5a and Fig.C.5b. In Fig.C.5a there is sufficient evidence to reject the null hypotheses pertaining to both individual ROIs in *post hoc* analysis. However, in Fig.C.5a there is only sufficient evidence to reject the null hypothesis pertaining to the first ROI.

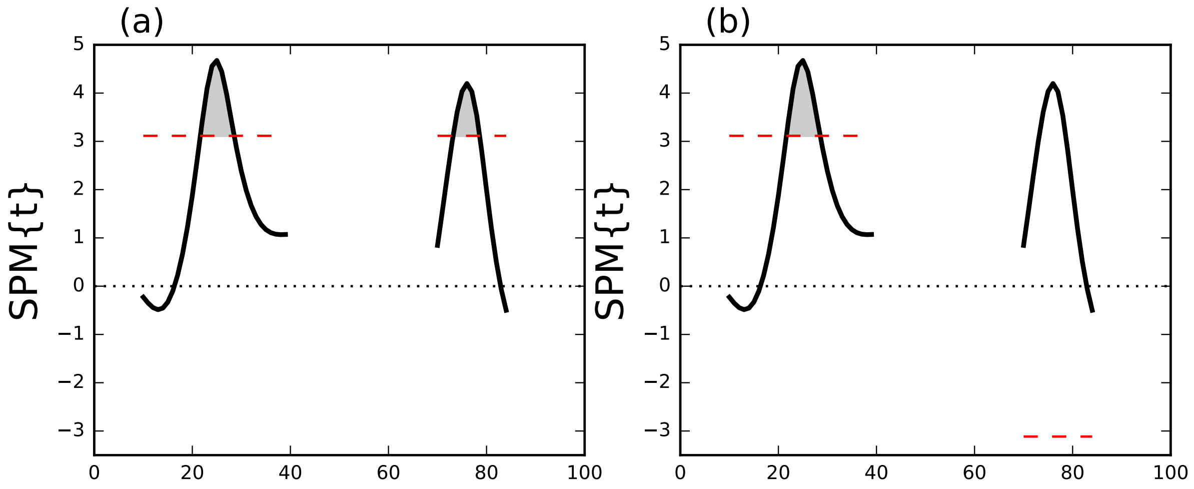

Figure C.5: Example directional ROI analysis. (a) Both ROIs are positive-directed. (b) The first and second ROIs are positive- and negative-directed, respectively.

Appendix D   Accessing datasets

All datasets analyzed in this paper are available in the **spm1d** software package (www.spm1d.org) and can be accessed via the **spm1d.data** interface as described below. Python and MATLAB code snippets appear in green and orange font, respectively.

All datasets in **spm1d.data** are organized as follows:

```
spm1d.data.XXXX.ZZZZ.DatasetName
```

where XXXX refers to the data modaility (e.g. univariate, one-dimensional), ZZZZ refers to the original or appropriate statistical test (e.g. one-sample t test), and DatasetName is a unique name assigned to each dataset, containing the main author's family name and publication year where appropriate.

Datasets A and B (Pataky et al., 2015)

Datasets A and B contain univariate, one-dimensional data ("uv1d"), the appropriate test for both is a one-sample t-test ("'t1"), and the dataset names are "SimulatedPataky2015a" and "Simulated-Pataky2015b". They can be accessed as indicated below. The YA and YB variables are both (10 x 101) arrays, where 10 is the number of responses and 101 is the number of time nodes used to approximate the continuum.

```
import spm1d
datasetA = spm1d.data.uv1d.t1.SimulatedPataky2015a()
datasetB = spm1d.data.uv1d.t1.SimulatedPataky2015b()
YA = datasetA.Y
YB = datasetB.Y
```

```
datasetA   = spm1d.data.uv1d.t1.SimulatedPataky2015a();
datasetB   = spm1d.data.uv1d.t1.SimulatedPataky2015b();
YA         = datasetA.Y;
YB         = datasetB.Y;
```

Dataset C (Neptune et al., 1999)

Dataset C contains multivariate, one-dimensional data ("mv1d"), the appropriate test is a paired Hotellings T^2 test ("hotellings paired"), and the dataset name is "Neptune1999kneekin". They can be accessed as indicated below. The YA and YB variables are both (8 x 101 x 3), where 8 is the number of responses, 101 is the number of time nodes, and 3 is the number of vector components. Although

these data are multivariate, in this study univariate analyses were conducted on only the first vector component (variables yA and yB).

```
import spm1d
dataset    = spm1d.data.mv1d.hotellings_paired.Neptune1999kneekin()
YA,YB      = dataset.YA, dataset.YB
yA,yB      = YA[:,:,0], YB[:,:,0]
```

```
dataset     = spm1d.data.mv1d.hotellings_paired.Neptune1999kneekin();
[YA,YB]     = deal( dataset.YA, dataset.YB );
[YA,YB]     = deal( YA(:,:,1), YB(:,:,1) );
```

Dataset D (Pataky et al., 2008)

Dataset D contains univariate, one-dimensional data ("uv1d"), the appropriate test is one-way ANOVA ("anova1"), and the dataset name is "SpeedGRFcategorical". Individual subjects' data can be loaded using the "subj" keyword as indicated below. The Y and A variables in each dataset are (60 x 101), and (60 x 1), respectively, where 60 is the number of responses and 101 is the number of time nodes. Y contains the GRF data and A contains a vector of condition labels, where the labels "1", "2", and "3" refer to slow, normal and fast walking, respectively. Note that 20 repetitions of each condition were conducted and that the conditions were presented in a randomized order. In this paper only the normal and fast conditions were considered.

The Y00 and Y01 variables are both (20 x 101) and were used in the Stage 1 analyses (Fig.6a). The Y0 and Y1 variables are both (6 x 101) and contain within-subject mean trajectories for each of the six subjects, and were used in the State 2 analyses (Fig.6b–c).

```
# load pilot subject's data:
subj0    = 0
dataset  = spm1d.data.uv1d.anova1.SpeedGRFcategorical(subj=subj0)
Y,A      = dataset.Y, dataset.A
Y00      = Y[A==2]
Y01      = Y[A==3]

# load experiment subjects' data:
SUBJ    = [2, 3, 4, 5, 6, 7]
Y0      = []
Y1      = []
for subj in SUBJ:
    dataset    = spm1d.data.uv1d.anova1.SpeedGRFcategorical(subj=subj)
    Y,A        = dataset.Y, dataset.A
    Y0.append( Y[A==2].mean(axis=0) )
    Y1.append( Y[A==3].mean(axis=0) )
Y0,Y1      = np.array(Y0), np.array(Y1)
```

```
% load pilot subject's data:
subj0     = 0;
dataset   = spm1d.data.uv1d.anova1.SpeedGRFcategorical(subj0);
[Y,A]     = deal( dataset.Y, dataset.A );
Y00       = Y(A==2,:);
Y01       = Y(A==3,:);

% load experiment subjects' data:
SUBJ      = [2 3 4 5 6 7];
Y0        = zeros(6, 101);
Y1        = zeros(6, 101);
for i = 1:6
    dataset   = spm1d.data.uv1d.anova1.SpeedGRFcategorical( SUBJ(i) );
    [Y,A]     = deal( dataset.Y, dataset.A );
    Y0(i,:)   = mean( Y(A==2,:), 1);
    Y1(i,:)   = mean( Y(A==3,:), 1);
end
```

References

Adler, R. J. and Taylor, J. E. (2007). *Random Fields and Geometry*. Springer-Verlag.

Brett, M., Anton, J. L., Valabregue, R., and Poline, J. B. (2002). Region of interest analysis using the marsbar toolbox for spm 99. *NeuroImage*, 16(2):S497.

Friston, K. J., Ashburner, J. T., Kiebel, S. J., Nichols, T. E., and Penny, W. D. (2007). *Statistical Parametric Mapping: The Analysis of Functional Brain Images*. Elsevier/Academic Press, Amsterdam.

Neptune, R. R., Wright, I. C., and van den Bogert, A. J. (1999). Muscle coordination and function during cutting movements. *Medicine & Science in Sports & Exercise*, 31(2):294–302, data: `http://isbweb.org/data/rrn/`.

Pataky, T. C., Caravaggi, P., Savage, R., and Crompton, R. H. (2008). Regional peak plantar pressures are highly sensitive to region boundary definitions. *Journal of Biomechanics*, 41(12):2772–2775.

Pataky, T. C., Vanrenterghem, J., and Robinson, M. A. (2015). Zero- vs. one-dimensional, parametric vs. non-parametric, and confidence interval vs. hypothesis testing procedures in one-dimensional biomechanical trajectory analysis. *Journal of Biomechanics*, 48(7):1277–1285.

Pataky, T. C., Vanrenterghem, J., and Robinson, M. A. (2016). The probability of false positives in zero-dimensional analyses of one-dimensional kinematic, force and emg trajectories. *Journal of Biomechanics*, 49:1468–1476.
